# Science and Mercy

**Published:** 1914-03

**Authors:** 


### OUR CONTEMPORARIES

### Science and Mercy

The antivivisectionists have been putting out a circular in Philadelphia, with the statement that Dr. George W. Crile made experiments on 148 dogs "in an endeavor to learn the extent of the agony that can be inflicted on a living animal." Do the kind-hearted women who are backing this movement believe that Dr. Crile did anything of the sort? When they leave out all mention of anesthesia, do they do it by accident? If not by accident, why do they do it? Surgeons until recently thought that when a patient was unconscious they could tear loose adhesions and manipulate tissues roughly without doing mischief. Crile's experiments were to determine whether this view was correct. He found that it was not; that serious injury could be caused by shock even when there was no consciousness. Realizing the difference between psychic shock, which is prevented by anesthesia, and traumatic shock, which is not prevented by anesthesia, is an important step ahead, which has already resulted in a lower death rate and a shorter time for recovery. Crile, like other men of science who are called monsters of cruelty, by these kind but ignorant sentimentalists, is the apostle of gentleness.—*Harper's Weekly.*

The foregoing, appearing in a medical journal, would not impress us particularly, as a professional journal is expected both to appreciate the value of Crile's work and to favor animal experimentation. Coming from a high class popular periodical, it will have an influence over lay judgment that no medical journal could exert.

---

The *Medical Council,* of February, contains an interesting editorial suggesting light starvation as a cause of cancer and citing the rarity of cancer among savages, in corroboration of this theory.

---

Dr. Albert Vander Veer of Albany has an interesting article on the Study of Medicine, Fifty Years Ago and Today, in the *Quarterly* of the Federation of State Medical Boards.

---

Dr. Charles Wardell Stiles of the U. S. Public Health Service has lately written an article in which, from experience at various medical meetings, and in physicians' offices, he concludes that the principles of hygiene or even of decency, have not permeated the medical profession. The *Memphis Medical Monthly,* January, 1914, reprints his article and strenuously denies its allegations as typic of the profession.

---

The *International Hospital Record,* formerly a monthly, for the last seven months, a weekly, will in the future appear semi-monthly. The editor candidly states that the labor of issuing every week is too great.

